# Supplementary material for: Structural basis of cell wall anchoring by SLH domains in Paenibacillus alvei
Source: Nat Commun. 2018 Aug 7;9:3120. doi: 10.1038/s41467-018-05471-3 (PMC6081394; doi:10.1038/s41467-018-05471-3)
Supplement: Supplementary file 1 — Supplementary Information [file 41467_2018_5471_MOESM1_ESM.pdf]

Supplementary Information for

Structural basis of cell wall anchoring by SLH domains in *Paenibacillus alvei*

Blackler *et al.*

This PDF includes:

Supplementary Methods

Supplementary Note 1

Supplementary Figures 1-9

Supplementary Tables 1-3

Supplementary References

Supplementary Methods

General synthetic methods. All purchased chemicals were used without further purification unless stated otherwise. Solvents (CH_2Cl_2, DMF, pyridine) were dried over activated 4 Å molecular sieves. Dry MeOH (secco solv) was purchased from Merck. Compound **1** and donor **9** were prepared according to literature [1,2]. Concentration of organic solutions was performed under reduced pressure <40°C. Optical rotations were measured with a Perkin-Elmer 243 B Polarimeter. $[\alpha]_D^{20}$ values are given in units of 10-1 deg cm^2 g^{-1}. Thin layer chromatography was performed on Merck precoated plates: generally on 5 x 10 cm, layer thickness 0.25 mm, Silica Gel $60F_{254}$; alternatively on HP-TLC plates with 2.5 cm concentration zone (Merck). Spots were detected by dipping reagent (anisaldehyde-H_2SO_4) and heating. For column chromatography silica gel (0.040–0.063 mm) was used. HP-column chromatography was performed on pre-packed columns (YMC-Pack SIL-06, 0.005 mm, 250 x 10 mm and 250 x 20 mm). Size exclusion chromatography was performed on Bio-Gel® P-2 Gel extra fine < 45 µm (wet) (1 x 30 cm). NMR spectra were recorded with a Bruker Avance III 600 instrument (600.22 MHz for ^1H, 150.93 MHz for ^{13}C) using standard Bruker NMR software. ^1H NMR spectra were referenced to 7.26 ($CDCl_3$) and 0.00 (D_2O, external calibration to 2,2-dimethyl-2-silapentane-5-sulfonic acid) ppm. ^{13}C NMR spectra were referenced to 77.00 ($CDCl_3$) and 67.40 (D_2O, external calibration to 1,4-dioxane) ppm. ESI-MS data were obtained on a Waters Micromass Q-TOF Ultima Global instrument.

Synthesis of mono- and disaccharide ligands. The synthesis of the ligands **8** and **12** is shown in Supplementary Fig. 2. The monosaccharide ligand **8** was prepared from known methyl glucoside **1** [1]. Inversion of configuration at position 2 and introduction of the azide group was achieved by reaction of an intermediate 2-*O*-triflate and subsequent treatment with sodium azide in DMF in 84% yield. Staudinger reduction of the azide with triphenylphosphine afforded the 2-deoxy-2-amino-mannopyranoside **3**. Chromatographic removal of the phosphine-derived products, however, could not be fully accomplished. Hence polymer-bound triphenylphosphine was used. Repeated hydrolysis of the resin was needed in order to fully recover product **3**. *N*-Acetylation of **3** giving **4** and subsequent hydrolysis of the benzylidene group with trifluoroacetic acid proceeded smoothly to afford the 3-*O*-benzyl glycoside **5**. Introduction of the pyruvic acid ketal was achieved by reaction of **5** with methyl pyruvate in the presence of TMSO-triflate [3], which gave the *S*-configured pyruvyl derivative **6** (53%) and the crystalline debenzylated product **7** (33%). The *S*-configuration of the stereogenic center of the pyruvic acetal was assigned on the basis of the characteristic ^1H and ^{13}C NMR chemical shifts [4]. Hydrogenation of **6** with 10% Pd-C in methanol gave compound **7** which was treated with aqueous NaOH and subjected to purification on BioGel-P2 to give the monosaccharide ligand **8** as sodium salt in 91% yield.

Compound **7** served as glycosyl acceptor in a TMSO-triflate promoted coupling with the *N*-Troc-protected trichloroacetimidate donor **9** [2] in MeCN to afford the β-(1→3)-linked disaccharide derivative **10** in modest yield (32%) with additional byproduct formation and with recovery of unreacted acceptor **7** (19%). The low reactivity of pyruvyl-substituted glycosyl acceptors has also been observed in the literature [3]. Troc-removal by reaction with Zn-dust was followed by *N*-acetylation to produce disaccharide **11** in 82% yields. Despite several attempts by HPLC-separations, product **11** could not be fully purified and still contained approximately 7% of an unknown constituent. Zemplén-de-*O*-acetylation and alkaline hydrolysis afforded the target disaccharide ligand **12** in 87% yield. NMR spectra of ligands **8** and **12** are shown in

Supplementary Figs. 3 and 4. Methyl 2-azido-3-*O*-benzyl-4,6-*O*-benzylidene-2-deoxy-β-D-*manno*-pyranoside (2). Compound **1** (298 mg, 0.80 mmol) was co-evaporated twice with toluene before dry DCM (5.14 mL) and dry pyridine (2.57 mL) were added. The solution was stirred at 0°C under Arand triflic anhydride (0.326 mL, 1.936 mmol) was added. After 45 min additional triflic anhydride (0.050 mL, 0.297 mmol) was injected into the solution as TLC showed incomplete conversion. The reaction was stopped after 75 min. The mixture was diluted with DCM, washed with saturated aq $NaHCO_3$, dried ($MgSO_4$) and concentrated. The residue was dissolved in anhydrous DMF (8.57 mL). Sodium azide (260.1 mg, 4.00 mmol) was added and the reaction mixture was stirred for 21 h at 70°C. The off-white solid was removed by filtration of the reaction mixture over Celite. The filtrate was concentrated and the residue was suspended in DCM. The suspension was filtered once more over Celite and the yellow filtrate was concentrated. Purification of the crude product by column chromatography (toluene-EtOAc 6:1) gave the azido-derivative **2** as light yellow syrup. Yield: 269 mg (84%); R_f 0.61 (toluene-EtOAc 6:1); ^{1}H-NMR (600 MHz, $CDCl_3$):δ 7.50-7.48 and 7.41-7.25 (m, 10H, 1xCH*Ph* and 1xOCH$_2$*Ph*), 5.60 (s, 1H, C*H*Ph), 4.89 and 4.75 (2d, 2H, ^2J12.4 Hz, OCH_2Ph), 4.46 (d, 1H, $^3J_{1,2}$1.4 Hz, H-1), 4.32 (dd, 1H, $^3J_{5,6a}$ 5.0 Hz,$^2J_{6a,6b}$10.5 Hz, H-6a), 4.04 (app. t, 1H, $^3J_{3,4}$=$^3J_{4,5}$9.5 Hz, H-4), 3.98 (dd, 1H, $^3J_{2,3}$3.7 Hz, H-2), 3.88 (app. t, 1H, H-6b), 3.75 (dd, 1H, H-3), 3.54 (s, 3H, OMe), 3.46 (ddd, 1H, $^3J_{5,6b}$10.0 Hz, H-5) ppm. The chemical shifts agree with published data [5].

Methyl 2-acetamido-3-*O*-benzyl-4,6-*O*-benzylidene-2-deoxy-β-D-*manno*-pyranoside (4). Compound **2** (787.0 mg, 1.980 mmol) and polymer-bound PPh_3 (1.6 mmol g^{-1} resin, 100-200 mesh, 3.094 g, 4.951 mmol) were treated with dry DCM (26.0 mL). The mixture was stirred at RT under Ar for 18 h. The polymer was filtered and washed several times with EtOAc, DCM and MeOH. Concentration of the filtrate gave a low amount of the intermediate amine 3 (50 mg). The polymer resin was then suspended in CH_3CN (20.0 mL), H_2O (600 µL, 33.3 mmol) was added and the mixture was stirred for 30 min, which gave 20 mg of 3. The polymer was then further treated with THF (20.0 mL) and H_2O (360 µL, 20.0 mmol) for 1 h at 55°C and for 15 h at RT. The polymer was washed with EtOAc several times and concentration of the combined filtrates gave the amine 3 (550 mg). Hydrolysis of the polymer was continued under the same conditions and stirring for 2 h at 55 °C, which provided an additional amount of the 3 (120 mg). An aliquot of 3 (650 mg, 1.75 mmol) was dissolved in pyridine (2.0 mL) and the solution was treated with acetic anhydride (1.3 mL, 13.8 mmol) for 105 min at RT under Ar. MeOH was added at 0°C, the solution was concentrated *in vacuo* and co-evaporated once with toluene. A second portion of the intermediate 3 (122 mg, 0.328 mmol) was acetylated with pyridine (1.5 mL) and Ac_2O (0.5 mL, 5.29 mmol) for 30 min and processed as described. The combined acetylated crude products were purified by MPLC (EtOAc-toluene 3:1 →EtOAc, flow rate: 60 to 50 mL min^{-1}) which afforded 4 as a yellow syrup (615 mg, 75%); R_f0.42 (EtOAc, HPTLC); $[\alpha]_D^{20}$ - 62.9 (*c* 1.0, $CHCl_3$). ^{1}H-NMR (600 MHz, $CDCl_3$):δ 7.50-7.48 and 7.41-7.29 (m, 10H, 1xCH*Ph* and 1xOCH$_2$*Ph*), 5.76 (d, 1H, $^3J_{NH,2}$9.6 Hz, N*H*), 5.59 (s, 1H, C*H*Ph), 4.88 (ddd, 1H, $^3J_{1,2}$1.9, $^3J_{2,3}$ 4.1, $^3J_{NH,2}$9.6 Hz, H-2), 4.81 and 4.66 (2d, 2H, ^2J 12.2 Hz, OCH_2Ph), 4.53 (d, 1H, H-1), 4.35 (dd, 1H, $^3J_{5,6a}$ 4.9, $^2J_{6a,6b}$10.4 Hz, H-6a), 3.80 (app. t, 1H, $^3J_{5,6b}$=$^2J_{6a,6b}$10.3 Hz, H-6b), 3.79-3.75 (m, 2H, H-3 and H-4), 3.50 (s, 3H, OMe), 3.46 (dt, 1H, $^3J_{4,5}$=$^3J_{5,6b}$9.7 Hz, H-5), 2.08 (s, 3H, CH_3CO) ppm. ^{13}C-NMR (150 MHz, $CDCl_3$):δ 170.83 (s, CH$_3$*C*O), 137.93 and 137.35 (2s, 1xCH*Ph* and 1xOCH$_2$*Ph*), 129.16-126.18 (10d, 5xCH*Ph* and 5xOCH$_2$*Ph*), 101.76 (d, *C*HPh), 101.48 (d, C-1), 79.06 and 75.63 (2d, C-3 and C-4), 71.80 (t, O*C*H$_2$Ph), 68.92 (t, C-6), 67.16 (d, C-5), 57.15 (q, OMe), 50.03 (q, *C*H$_3$CO) ppm. HR-MS: $[M+Na]^+$ *m/z* calculated: 436.1731; found: 436.1715.

Methyl 2-acetamido-3-*O*-benzyl-2-deoxy-β-D-*manno*-pyranoside (5). Compound **4** (605.9 mg, 1.465 mmol) was dissolved in dry DCM (20 ml). Trifluoroacetic acid (12.1 mL, 157.32 mmol) was added to the solution at 0°C under Ar and the solution was stirred for 1 h. The solution was concentrated and the resulting brown residue was co-evaporated twice with toluene. Purification by column chromatography (EtOAc-toluene 3:1 → EtOAc-EtOH 4:1) furnished unreacted **4** (173 mg, 28.5%) followed by **5** as syrup (296 mg, 62%); R_f 0.33 (EtOAc-EtOH 9:1, HPTLC);$[\alpha]_D^{23}$ – 44.6 (c 0.75, MeOH). ^{1}H-NMR (600 MHz, MeOH-d_4): δ 7.40-7.38, 7.32-7.29 and 7.27-7.24 (m, 5H, OCH_2Ph), 4.82 and 4.48 (2d, 2H, ^2J 11.1 Hz, OCH_2Ph), 4.74 (dd, 1H, $^3J_{1,2}$ 1.5, $^3J_{2,3}$ 4.2 Hz,H-2), 4.52 (d, 1H, H-1), 3.86 (app. d, 2H, $^3J_{5,6a}$=$^3J_{5,6b}$ 3.4 Hz, H-6a and H-6b), 3.61 (app. t, 1H, $^3J_{3,4}$=$^3J_{4,5}$9.7 Hz, H-4), 3.75 (dd, 1H, H-3), 3.48 (s, 3H, OMe), 3.26 (dt,1H, $^3J_{4,5}$9.8Hz, H-5), 2.01 (s, 3H, CH_3CO) ppm. ^{13}C-NMR (150 MHz, MeOH-d_4): 173.87 (s, CH_3CO), 139.64 (s, OCH_2Ph), 129.22 and 128.60 (5d, OCH_2Ph), 102.02 (d, C-1), 81.60 (d, C-3), 78.18 (d, C-5), 72.20 (t, OCH_2Ph), 67.04 (d, C-4), 61.95 (t, C-6), 57.00 (q, OMe), 50.88 (d, C-2), 22.60 (q, CH_3CO) ppm. HR-MS: $[M+H]^+$ *m/z* calculated: 326.1598; found: 326.1599.

Methyl 2-acetamido-3-*O*-benzyl-2-deoxy-4,6-*O*-[1-(methoxycarbonyl)ethylidene]-β-D-*manno*-pyranoside (6). Methyl pyruvate (190 µL, 2.108 mmol) and TMSO-triflate (382 µL, 2.108 mmol) were added in succession to a stirred solution of compound **5** (298.2 mg, 0.917 mmol) in CH_3CN (3 mL) at RT. Stirring was continued for 105 min when TLC showed formation of undesired side products. The solution was concentrated, diluted with EtOAc, neutralized with Et_3N and concentrated. The crude mixture was purified by column chromatography (EtOAc-toluene 2.5:1 to EtOAc-EtOH 9:1) which afforded **6** (198.7 mg, 53%) followed by debenzyl derivative **7** (97 mg, 33%). For preparation of an analytically pure sample, aliquots of compound **6** were purified by column chromatography (EtOAc-toluene 5:1) and by HPLC (EtOAc: toluene 6:1, column: YMC 250x20, flow rate: 15 mL/min), respectively; R_f 0.74 (EtOAc-EtOH 9:1, HPTLC);$[\alpha]_D^{23}$ – 35.0 (*c* 0.98, $CHCl_3$).^{1}H-NMR (600 MHz, $CDCl_3$): δ 7.41-7.39, 7.35-7.33 and 7.29-7.27 (m, 5H, OCH_2Ph), 5.76 (d, 1H, $^3J_{NH,2}$9.5 Hz, N*H*), 4.77 (app. s, 2H, OCH_2Ph), 4.73 (dd, 1H, $^3J_{1,2}$ 2.0, $^3J_{2,3}$5.5 Hz,H-2), 4.47 (d, 1H, H-1), 4.08 (dd, 1H, $^3J_{5,6a}$5.0, $^2J_{6a,6b}$10.7 Hz, H-6a), 3.84 (s, 3H, CO_2Me), 3.72 (app. t, 1H, $^3J_{4,5}$9.2 Hz, H-4), 3.75 (app. t, 1H, $^3J_{5,6b}$10.7 Hz, H-6b), 3.74 (dd, 1H, $^3J_{3,4}$8.5 Hz, H-3), 3.42 (s, 3H, OMe), 3.38 (m, 1H, H-5), 2.03 (s, 3H, CH_3CO), 1.57 (s, 3H, Me) ppm. ^{13}C-NMR (150 MHz, $CDCl_3$): δ 170.49 and 170.42 (2s*, CH_3CO), 170.13 (s, $MeCCO_2Me$), 138.04 (s, OCH_2Ph), 100.82 (d, C-1), 99.15 (s, $MeCCO_2Me$), 75.81 (d, C-4), 74.52 (d, C-3), 71.44 (t, OCH_2Ph), 65.94 (d, C-5), 65.54 (t, C-6), 56.62 (q, OMe), 52.76 (q, $MeCCO_2Me$), 49.36 and 49.28 (2d*, C-2), 25.61 (q, $MeCCO_2Me$), 23.47 and 23.42 (2q*, CH_3CO) ppm. * Signal duplication was observed.HR-MS: $[M+Na]^+$ *m/z* calculated: 432.1629; found: 432.1627.

Methyl 2-acetamido-2-deoxy-4,6-*O*-[1-(methoxycarbonyl)ethylidene]-β-D-*manno*-pyranoside (7). A solution of **6** (87.0 mg, 0.212 mmol) in dry MeOH was hydrogenated at atmospheric pressure in the presence of 33.6 mg 10% Pd-C for 5.5 h at RT. The suspension was filtered over Celite and the filtrate was concentrated. The product **7** was partly crystallized in a mixture of *n*-hexane and EtOAc. The remaining mother liquor was concentrated and purified by column chromatography (EtOAc-MeOH 95:5). Combined yield for **7**: 44.7 mg (66%). Colorless crystals, m.p. 192-195 °C; R_f 0.29 (EtOAc-EtOH 9:1, HPTLC), $[\alpha]_D^{20}$ – 49.9 (*c* 0.62, MeOH). ^{1}H-NMR (600 MHz, MeOH-d_4): δ 4.58 (d, 1H, $^3J_{1,2}$ 1.9 Hz, H-1), 4.54 (dd, 1H, $^3J_{2,3}$ 4.7 Hz,H-2), 3.99 (dd, 1H, $^3J_{5,6a}$ 5.0 Hz, $^2J_{6a,6b}$ 10.5 Hz, H-6a), 3.83 (dd, 1H, $^3J_{3,4}$ 9.7 Hz, H-3), 3.83 (s, 3H, CO_2Me), 3.78 (app. t, 1H, $^3J_{5,6b}$ = $^2J_{6a,6b}$ 10.5 Hz, H-6b), 3.55 (app. t, 1H, $^3J_{3,4}$ = $^3J_{4,5}$ 9.7 Hz, H-

4), 3.44 (s, 3H, OMe), 3.31 (m, 1H, H-5), 2.01 (s, 3H, C*H₃*CO), 1.48 (s, 3H, Me) ppm.^{13}C-NMR (150 MHz, MeOH-d_4)*: δ 174.77 (s, CH_3*C*O), 172.10 (s, MeC*C*O_2Me), 102.60 (d, C-1), 100.70 (s, Me*C*CO_2Me), 76.31 (d, C-4), 71.06 (d, C-3), 68.29 (d, C-5), 65.93 (t, C-6), 57.17 (q, OMe), 54.72 (q, MeCCO_2*Me*), 53.17 (d, C-2), 25.92 (q, *Me*CCO_2Me), 22.64 (q, *C*H_3CO) ppm. HR-MS: $[M+H]^+$ *m/z* calculated: 320.1340; found: 320.1340.

*Note: ^{13}C NMR assignments are in contrast to the published assignments of the terminal 4,6-O-pyruvyl-β-ManNAc residue in the trisaccharide 4,6-O-pyruvyl-β-ManNAc-(1→4)- -β-GlcNAc-(1→6)-α-GlcN-OR indicating that the shift assignments of the β-ManNAc and α-GlcN residue have to be interchanged [41].

Methyl 2-acetamido-2-deoxy-4,6-*O*-[1-carboxyethylidene]-β-D-*manno*-pyranoside sodium salt (8). A solution of 0.2 M NaOH (1.5 mL) was added to compound **7** (6.2 mg, 0.0194 mmol). The mixture was stirred at RT for 3 h 15 min. Dowex AG1X cation-exchange resin (H^+-form) was added until pH 7.5. The suspension was filtered and the filtrate was lyophilized. Product **8** was desalted by size exclusion chromatography using a BioGel P2-column (5% aq EtOH) and the product fraction was lyophilized. Yield: 5.8 mg of **8** as a colorless solid (91%); R_f0.94 ($CHCl_3$-MeOH-H_2O 10:10:3);$[\alpha]_D^{20}$ – 34.2 (*c* 0.51, H_2O). ^{1}H-NMR (600 MHz, D_2O): δ 4.71 (d, 1H, $^3J_{1,2}$1.9 Hz, H-1), 4.51 (dd, 1H,$^3J_{2,3}$4.6 Hz,H-2), 4.01 (dd, 1H, $^3J_{5,6a}$5.0, $^2J_{6a,6b}$10.6 Hz, H-6a), 3.93 (dd, 1H, $^3J_{3,4}$10.1 Hz, H-3), 3.70 (app. t, 1H, $^3J_{5,6b}$=$^2J_{6a,6b}$10.6 Hz, H-6b), 3.58 (app. t, 1H, $^3J_{4,5}$9.9 Hz, H-4), 3.45 (s, 3H, OMe), 3.40 (ddd, 1H, H-5), 2.02 (s, 3H, C*H₃*CO), 1.43 (s, 3H, Me) ppm. ^{13}C-NMR (150 MHz, D_2O): δ 176.36 and 176.27 (2s, MeC*C*O_2Na and CH_3*C*O), 102.65 (s, Me*C*CO_2Na), 101.81 (d, C-1), 72.82 (d, C-4), 70.08 (d, C-3), 67.67 (d, C-5), 64.88 (t, C-6), 58.02 (q, OMe), 54.01 (d, C-2), 25.49 (q, *Me*CCO_2Na), 22.80 (q, *C*H_3CO) ppm. HR-MS: $[M+H]^+$ *m/z* calculated: 328.1003; found: 328.1008.

Methyl [3,4,5-tri-*O*-acetyl-2-deoxy-2-(2,2,2-trichloroethoxycarbonylamino)-β-D-*gluco*-pyranosyl]-(1→3)-2-acetamido-2-deoxy-4,6-*O*-[1-(methoxycarbonyl)ethylidene]-β-D-*manno*-pyranoside (10). A solution of predried donor **9** (151.1 mg, 0.242 mmol) in dry MeCN (6 mL) was added to acceptor **7** (38.6 mg, 0.121 mmol) and powdered acid-washed 4 Å molecular sieves, and the suspension was stirred under Ar at RT for 30 min. An aliquot of a 0.242 M TMS-triflate stock solution (100 µL, 0.0242 mmol) was added and stirring was continued for 1 h 45 min. Another aliquot of TMSO-triflate (100 µL, 0.0242 mmol) was added. TLC showed two products and some side products due to hydrolysis. The reaction was stopped after 2 h 50 min by adding a few drops of Et_3N. The suspension was filtered over Celite and the filtrate was concentrated. Purification of the residue by column chromatography (EtOAc-toluene 6:1 to EtOAc:EtOH 4:1) afforded crude **10** as a colorless syrup (30.2 mg, 32%), an unidentified by-product (9.5 mg) and unreacted acceptor **7** (7.3 mg, 19%). Final purification of **10** was achieved by HPLC (EtOAc to toluene 2:1, column: YMC 250x10, flow rate: 5 ml min^{-1}). R_f= 0.43 (EtOAc to toluene 6:1); $[\alpha]_D^{20}$ – 38.2 (*c*0.9, $CHCl_3$). ^{1}H-NMR (600 MHz, $CDCl_3$): δ 6.02 (d, 1H, $^3J_{NH,2'}$8.8 Hz, NH'), 5.87 (d, 1H, $^3J_{NH,2}$8.2 Hz, N*H*$COCH_3$), 5.23 (app. t, 1H, $^3J_{2',3'}$=$^3J_{3',4'}$9.8 Hz, H-3'), 5.12 (app. t, 1H, $^3J_{4',5'}$9.6 Hz, H-4'), 5.00 (d, 1H, $^3J_{1',2'}$8.4 Hz, H-1'), 4.85 and 4.59 (2d, 2H, ^2J=12.1 Hz, Cl_3CCH_2), 4.64 (m, 1H, H-2), 4.50 (d, 1H, $^3J_{1,2}$1.9 Hz, H-1), 4.16 (dd, 1H, $^3J_{5',6a'}$4.3,$^2J_{6a',6b'}$12.3 Hz, H-6a'), 4.21 (dd, 1H, $^3J_{5',6b'}$2.8 Hz, H-6b'), 4.08 (dd, 1H, $^3J_{5,6a}$5.0,$^2J_{6a,6b}$10.7 Hz, H-6a), 4.05 (dd, 1H, $^3J_{2,3}$ 4.7, $^3J_{3,4}$9.4 Hz, H-3), 3.87 (dt, 1H, H-2'), 3.80 (s, 3H, CO_2Me), 3.74 (app. t, 1H, $^3J_{5,6b}$10.7 Hz, H-6b), 3.73 (ddd, 1H, $^3J_{4',5'}$10.0 Hz, H-5'), 3.54 (app. t, 1H, $^3J_{4,5}$9.6 Hz, H-4), 3.46 (s, 3H, OMe), 3.38 (dt, 1H, H-5), 2.09-1.98 (4s, 12H, 1xNHCOC*H₃* and 3x$OCOCH_3$), 1.56 (s, 3H, Me) ppm. ^{13}C-NMR (150 MHz, $CDCl_3$): δ 172.43-

169.65 (5s, 3xO*C*OCH$_3$, 1xNH*C*OCH$_3$ and 1xNH*C*OOCH$_2$CCl$_3$), 170.23 (s, MeC*C*O$_2$Me), 154.69 (s, CH$_2$*C*Cl$_3$), 100.90 (d, C-1), 99.55 (s, Me*C*CO$_2$Me), 96.40 (d, C-1'), 74.56 (t, *C*H$_2$CCl$_3$), 73.70 (d, C-3), 73.37 (d, C-3'), 73.15 (d, C-4), 72.22 (d, C-5'), 69.09 (d, C-4'), 67.10 (d, C-5), 65.25 (t, C-6), 62.32 (t, C-6'), 57.12 (q, OMe), 55.91 (d, C-2'), 52.79 (q, MeCCO$_2$*Me*), 49.66 (d, C-2), 25.44 (q, *Me*CCO$_2$Me), 23.72-20.78 (4q, 3xOCO*C*H$_3$ and 1xNHCO*C*H$_3$) ppm. HR-MS: $[M+H]^+$ *m/z* calculated: 781.1387; found: 781.1371.Methyl (2-acetamido-3,4,5-tri-*O*-acetyl-2-deoxy-β-D-*gluco*-pyranosyl)-(1→3)-2-acet-amido-2-deoxy-4,6-*O*-[1-(methoxycarbonyl)ethylidene]-β-D-*manno*-pyranoside (11). Compound **10** (18.1 mg, 0.023 mmol) was dissolved in glacial acetic acid (2.0 ml) and Zn-powder (10 µm, 75.67 mg, 1.157 mmol) was added. The reaction mixture was stirred at RT under Ar for 18 h. As conversion was not complete, another portion of Zn-powder was added and stirring was continued for 1 h. The suspension was filtered over Celite and the solids were washed several times with glacial acetic acid. The combined filtrate was concentrated and co-evaporated three times with toluene. The off-white residue was dissolved in pyridine (1.2 mL) and acetic anhydride (600 µL, 6.347 mmol) was added and the solution was stirred for 105 min at RT. The reaction was quenched with MeOH (300 µl), solvents were evaporated and the residue was co-evaporated twice with toluene. Purification by column chromatography (EtOAc-MeOH 9.5:0.5 to EtOAc-MeOH 9:1) gave **11** containing a trace impurity (~7%) as a colorless amorphous solid (12.4 mg, 82%); R_f 0.35 (EtOAc-MeOH 9:1, HPTLC); $[\alpha]_D^{20}$ – 53.0 (*c* 0.92, MeOH).^{1}H-NMR (600 MHz, MeOH-d_4): δ 5.18 (app. t, 1H, $^3J_{2',3'}$=$^3J_{3',4'}$9.7 Hz, H-3'), 5.05 (app. t, 1H, $^3J_{4',5'}$9.6 Hz, H-4'), 4.82 (d, 1H, H-1'), 4.69 (dd, 1H, $^3J_{1,2}$ 1.6, $^3J_{2,3}$4.4 Hz,H-2), 4.56 (d, 1H, H-1), 4.33 (dd, 1H, $^3J_{5',6a'}$ 4.4, $^2J_{6a',6b'}$12.2 Hz, H-6a'), 4.18 (dd, 1H, $^3J_{5',6b'}$2.7 Hz, H-6b'), 4.08 (dd, 1H, $^3J_{3,4}$10.0 Hz, H-3), 3.99 (dd, 1H, $^3J_{5,6a}$5.0,$^2J_{6a,6b}$10.5 Hz, H-6a), 3.96 (dd, 1H, $^3J_{1',2'}$8.2 Hz, H-2'), 3.82 (m, 1H, H-5'), 3.81 (s, 3H, CO$_2$Me), 3.80 (app. t, 1H, $^3J_{5,6b}$10.6 Hz, H-6b), 3.66 (app. t, 1H, $^3J_{4,5}$9.8 Hz, H-4), 3.44 (s, 3H, OMe), 3.35 (dt, 1H, H-5), 2.06-1.98 (5s, 15H, 2xNHCOC*H$_3$* and 3xOCOCH$_3$), 1.48 (s, 3H, Me) ppm; ^{13}C-NMR (150 MHz, MeOH-d_4): δ 174.45-171.30 (5s, 3xO*C*OCH$_3$ and 2xNH*C*OCH$_3$), 171.91 (s, MeC*C*O$_2$Me), 102.50 (d, C-1), 100.74 (s, Me*C*CO$_2$Me), 98.32 (d, C-1'), 75.30 (d, C-3), 74.93 (d, C-3'), 74.18 (d, C-4), 73.08 (d, C-5'), 70.24 (d, C-4'), 68.59 (d, C-5), 65.86 (t, C-6), 63.36 (t, C-6'), 57.23 (q, OMe), 55.23 (d, C-2'), 53.10 (q, MeCCO$_2$*Me*), 51.28 (d, C-2), 25.87 (q, *Me*CCO$_2$Me), 23.05-20.60 (5q, 3xOCO*C*H$_3$ and 2xNHCO*C*H$_3$) ppm. HR-MS:$[M+Na]^+$ *m/z* calculated: 671.2270; found: 671.2266.

Methyl (2-acetamido-2-deoxy-β-D-*gluco*-pyranosyl)-(1→3)-2-acetamido-2-deoxy-4,6-*O*-(1-carboxyethylidene)-β-D-*manno*-pyranoside sodium salt (12). 0.1 M NaOMe-solution (1.0 mL) was added to a solution of compound **11** (4.5 mg, 0.007 mmol) in dry MeOH (2.0 mL) and stirred at RT for 80 min. The pH was adjusted to 7.0 by addition of Dowex H^+ ion exchange resin, the resin was filtered off and the filtrate was concentrated *in vacuo*. The residue was treated with 0.2 M NaOH-solution (1.0 mL) at RT for 3 h. The pH was adjusted to 7.5 by addition of Dowex H^+ ion exchange resin. The suspension was filtered and the filtrate was lyophilized. Purification by size exclusion chromatography on a BioGel-P2-column gave **12** as colorless amorphous solid. Yield: 3.2 mg (87%, containing ~7% impurity); R_f 0.32 (EtOAc-MeOH 1:1, HPTLC); $[\alpha]_D^{23}$ – 56.0 (*c* 0.37, H$_2$O). ^{1}H-NMR (600 MHz, D$_2$O): δ 4.63 (m, 2H, H-1 and H-2), 4.59 (d, 1H, $^3J_{1',2'}$8.5 Hz, H-1'), 4.17 (dd, 1H, $^3J_{2,3}$ 4.8, $^3J_{3,4}$10.4 Hz, H-3), 3.99 (dd, 1H, $^3J_{5,6a}$5.0,$^2J_{6a,6b}$10.7 Hz, H-6a), 3.90 (dd, 1H, $^3J_{5',6a'}$ 2.2, $^2J_{6a',6b'}$12.4 Hz, H-6a'), 3.72 (m, 1H, H-4), 3.71 (m, 1H, H-6b), 3.70 (m, 1H, H-2'), 3.69 (m, 1H, H-6b'), 3.50 (dd, 1H, $^3J_{2',3'}$ 10.3, $^3J_{3',4'}$8.8 Hz, H-3'), 3.42 (m, 1H, H-5'), 3.43 (s, 3H, OMe), 3.39 (m, 1H, H-5), 3.38 (m, 1H, H-4'), 2.01 and 1.99 (2s, 6H, 2xNHCOC*H$_3$*), 1.43 (s, 3H, Me) ppm.^{13}C-NMR (150 MHz, D$_2$O): δ

176.09 (s, MeC*C*O_2Na), 175.56 and 175.12 (2s, 2xNH*C*OCH_3), 102.83 (s, Me*C*CO_2Na), 102.09 (d, C-1), 98.87 (d, C-1'), 76.78 (d, C-5'), 74.87 (d, C-3'), 74.79 (d, C-3), 73.03 (d, C-4), 70.63 (d, C-4'), 67.82 (d, C-5), 64.81 (t, C-6), 61.61 (t, C-6'), 58.06 (q, OMe), 56.14 (d, C-2'), 51.05 (d, C-2), 25.41 (q, *Me*CCO_2Na), 23.25 and 22.86 (2q, 2xNHCO*C*H_3) ppm. HR-MS: $[M+H]^+$ *m/z* calculated: 531.1797; found: 531.1796.

Abbreviations used are: Ac, acetyl; Ar, argon; Bn, benzyl; D_2O, deuterium oxide; DCM, dichloromethane; DMF, *N,N*-dimethylformamide; EtOAc, ethyl acetate; H_2, hydrogen; *J*, coupling constant; MeCN, acetonitrile; MPLC, medium pressure liquid chromatography; NaOMe, sodium methoxide; Pd, palladium; Ph, phenyl; Pyr, pyruvyl; TFA, trifluoroacetic acid; THF, tetrahydrofuran; TLC, thin layer chromatography; TMSOTf, trimethylsilyl trifluoromethanesulfonate; Troc, trichloroethoxycarbonyl; *wt*, wild type

Supplementary Note 1:

Biophysical Characterization. The far-UV CD spectra of recombinant $SpaA_{SLH}$ with and without 4,6-Pyr-β-D-ManNAcOMe exhibited minima at 208 and 222 nm, indicative of a high α-helical content, with no significant differences between them (Supplementary Fig. 7). This is consistent with the observed structural changes occurring in loop regions only and is in agreement with the overall helical conformation observed for $SpaA_{SLH}$ and as described for the truncated S-layer proteins Sap_{SLH} of *B. anthracis* [6,7] and $SbsB_{SLH}$ of *Geobacillus stearothermophilus* PV72/p2 [8].

A notable difference in thermal stability was observed between unliganded and liganded $SpaA_{SLH}$, with T_m increasing from 48.0°C to 52.0°C (Supplementary Fig. 7), suggesting that ligand binding is stabilizing $SpaA_{SLH}$. The truncated S-layer protein SbsC of *G. stearothermophilus* was also reported to experience substantial stabilization upon binding to its native SCWP [9].

The Conserved TRAE Motif is Critical to SLH Trimer Folding. Attempts to overexpress the mutants $SpaA_{SLH}/TAAA_{12}$ (where TRAE of SLH1 and TVEE of SLH2 are both mutated to TAAA) and $SpaA_{SLH}/TAAA_{13}$ (where TRAE of SLH1 and TRAQ of SLH3 are both mutated to TAAA) in *E. coli* resulted in insoluble protein. This is in contrast to the previous study by Janesch *et al.*[10] that produced these mutants of full-length SpaA. In that case, the solubility of the double and triple TAAA mutants may have been preserved in the context of full-length SpaA.

Similar to the insolubility of double TAAA mutants of $SpaA_{SLH}$ that we observe in this study, May *et al.* [11] reported significantly reduced solubility of *Thermoanaerobacterium thermosulfurigenes* XynA SLH domains relative to wild-type when various mutations were made to the conserved TRAE motifs. Although in that case, sufficient amounts of soluble mutant proteins were recovered to assay their ability to bind PG.

The reduced solubility of SLH domains upon mutation of residues within the conserved TRAE motifs indicates that in addition to making essential interactions in binding SCWP, these residues are critical for the proper folding of the SLH domain trimer. The basis for this is revealed in crystal structures, where these residues are observed to precisely interlock between SLH domains to form the SCWP binding grooves.

SpaA *O*-Glycosylation is Proximal to SCWP Binding Sites. SpaA is *O*-glycosylated on at least two known sites, Tyr47 and Tyr155, with an average of 23 repeating units of {→3)-β-D-Gal*p*-(1→4)-[α-D-Glc*p*-(1→6)]-β-D-Man*p*NAc-(1→} that are linked by the adaptor →3)-[GroA-2→ OP(=O)OH→4-β-D-Man*p*NAc-(1→4)]-α-L-Rha*p*-(1→3)-α-L-Rha*p*-(1→3)-α-L-Rha*p*-(1→3)-β-D-Gal*p*-(1→*O*-Tyr [10,12]. The crystal structures of $SpaA_{SLH}$ show that these Tyr residues are proximal to the SCWP binding sites in G1 and G2, where it is possible that their glycosylation would affect SCWP binding. Furthermore, Tyr47 is within the SLH1 44-54 loop that was observed in multiple conformations, and its glycosylation may influence loop stability and groove structure. G3 lacks a known glycosylation site.

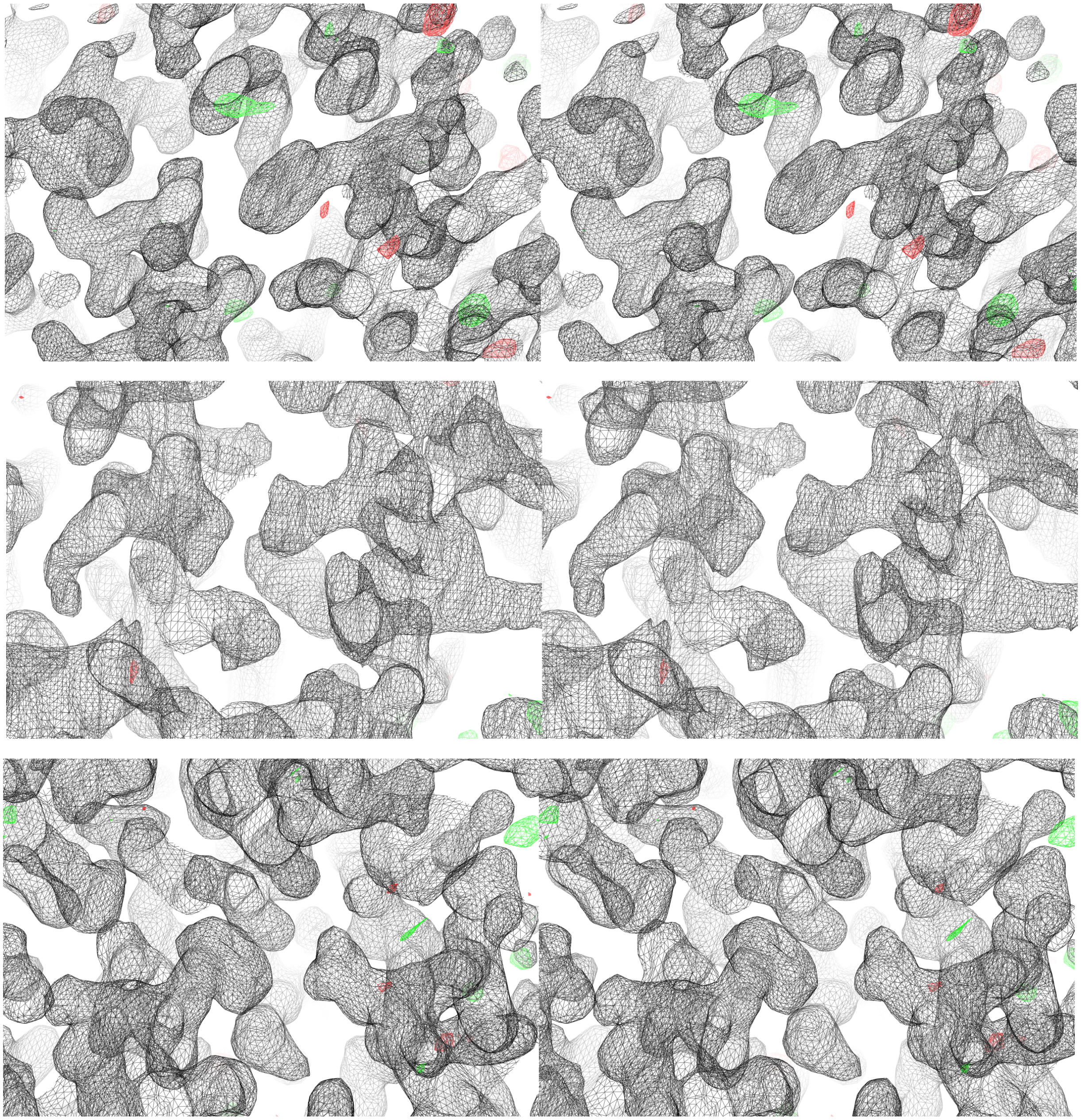

a

b

c

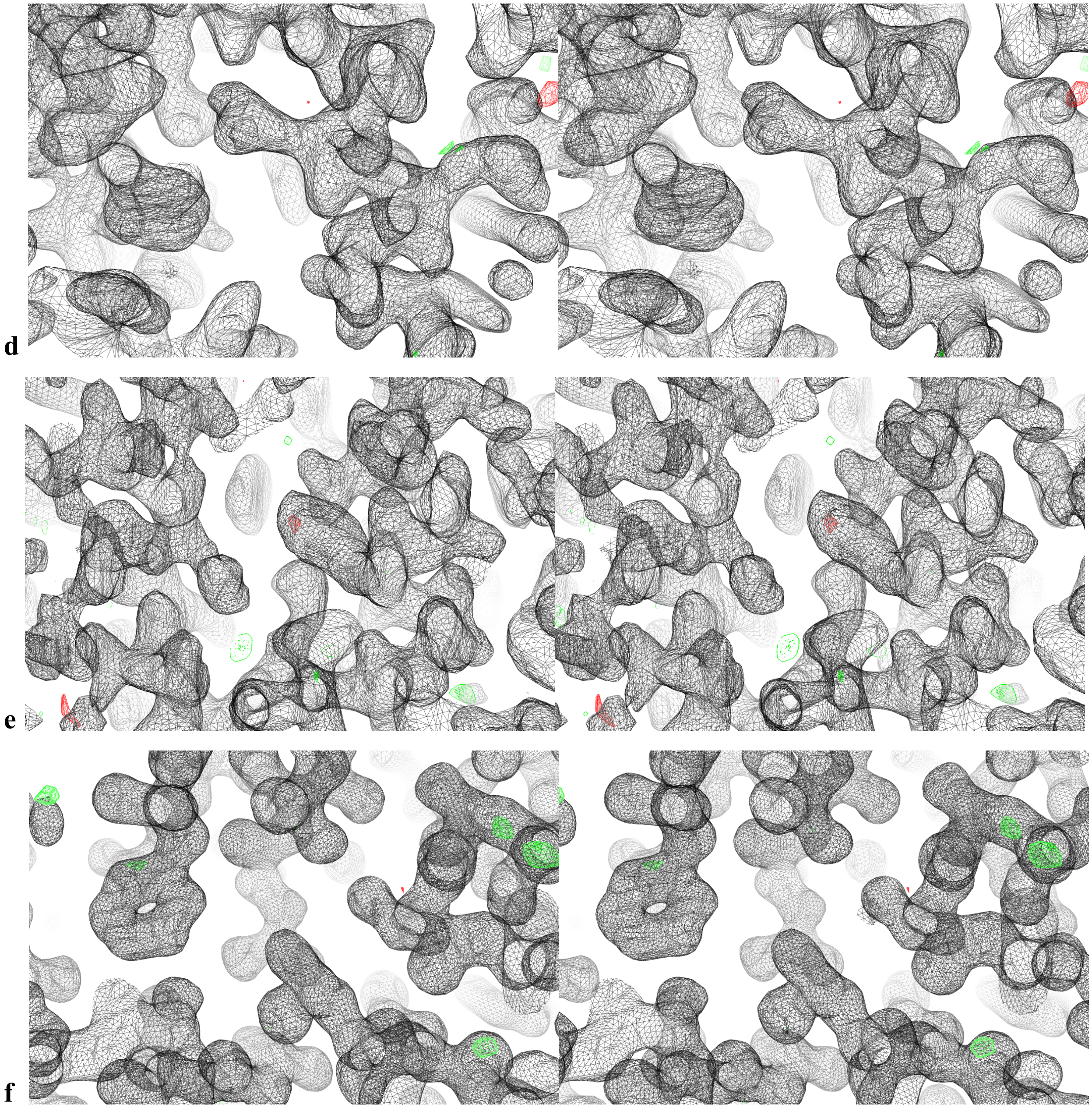

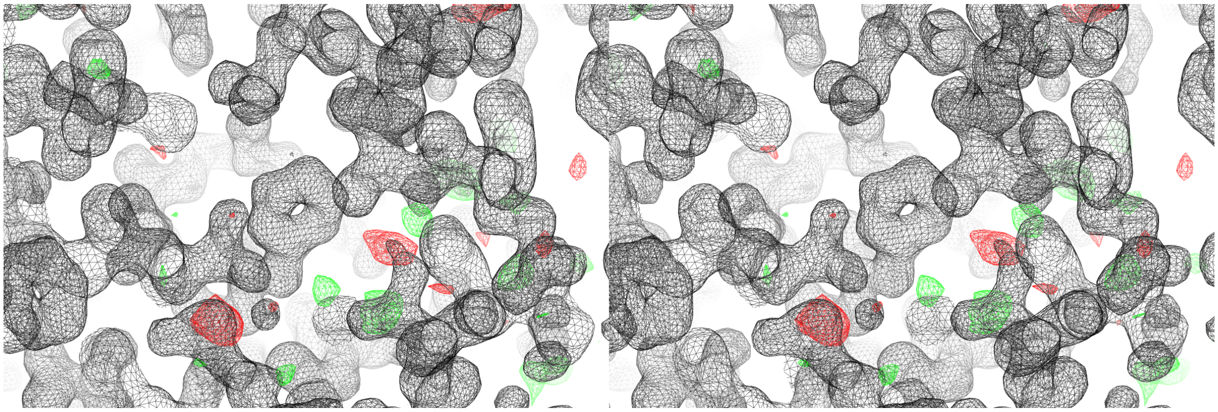

g

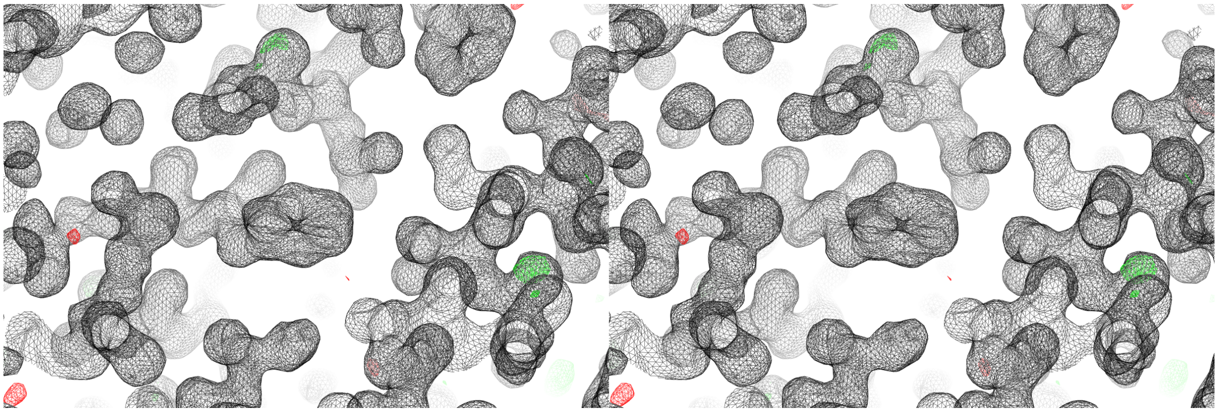

h

Supplementary Figure 1 | Representative electron density. Segments of electron density maps (2Fo-Fc map contoured to 1σ, black; Fo-Fc map contoured to 3σ, green, and -3σ, red) are shown for the structures presented in this work. **a**, 6CWC, **b**, 6CWF, **c**, 6CWH, **d**, 6CWI, **e**, 6CWL, **f**, 6CWM, **g**, 6CWN, **h**, 6CWR.

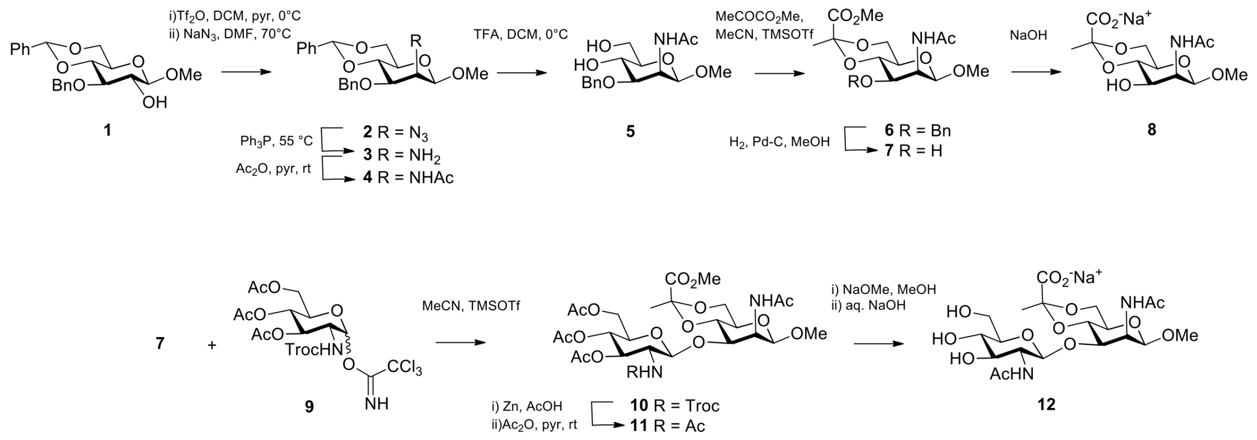

Supplementary Figure 2 | Synthetic reaction scheme. Reaction scheme is shown for the synthesis of 4,6-*O*-pyr-β-D-Man*p*NAcOMe (**8**) and β-D-Glc*p*NAc-(1→3)-4,6-*O*-pyr-β-D-Man*p*NAcOMe (**12**).

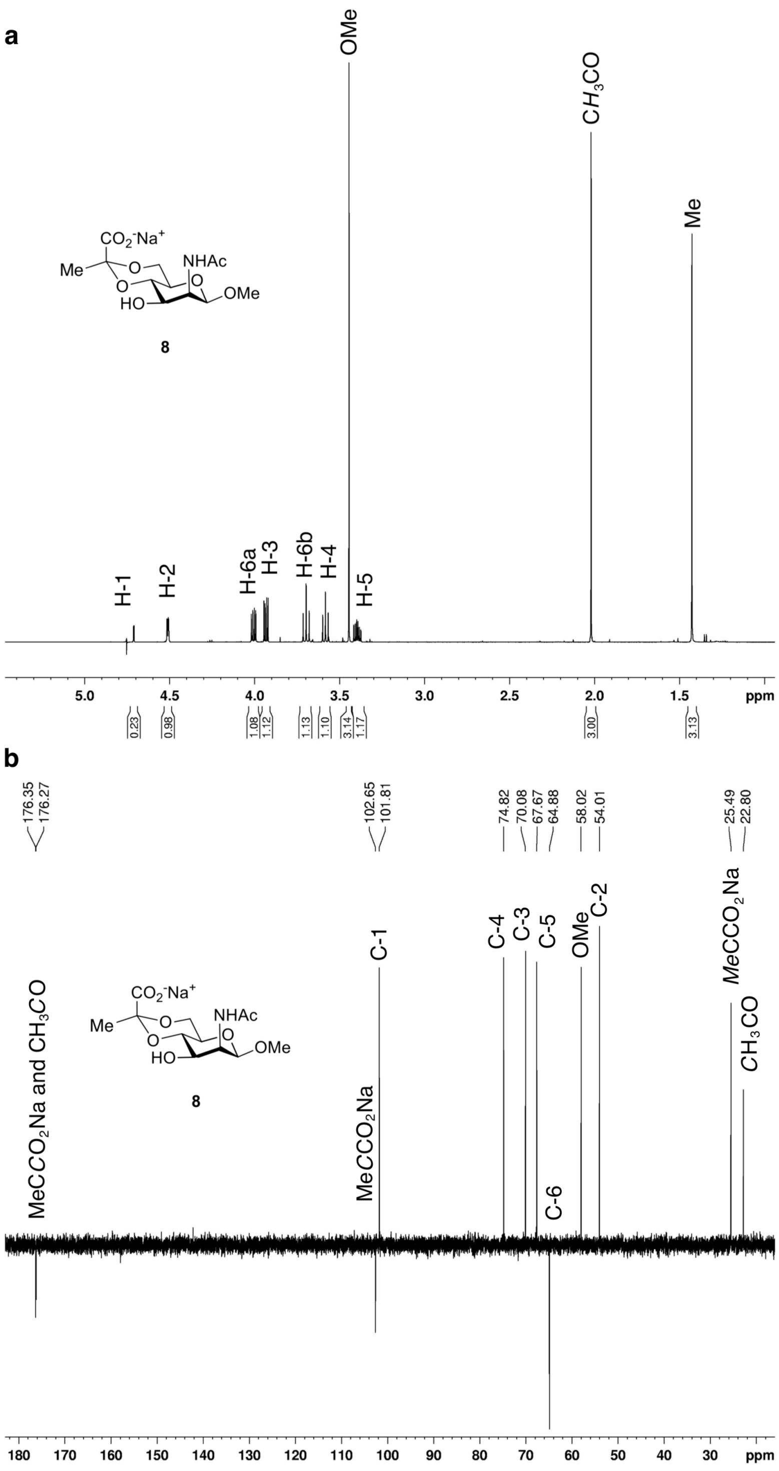

Supplementary Figure 3 | NMR spectra for compound 8. a, ^{1}H NMR spectrum (600 MHz) and **b,**^{13}C NMR spectrum (150 MHz) of 4,6-*O*-pyr-β-D-Man*p*NAcOMe (**8**) in D_2O.

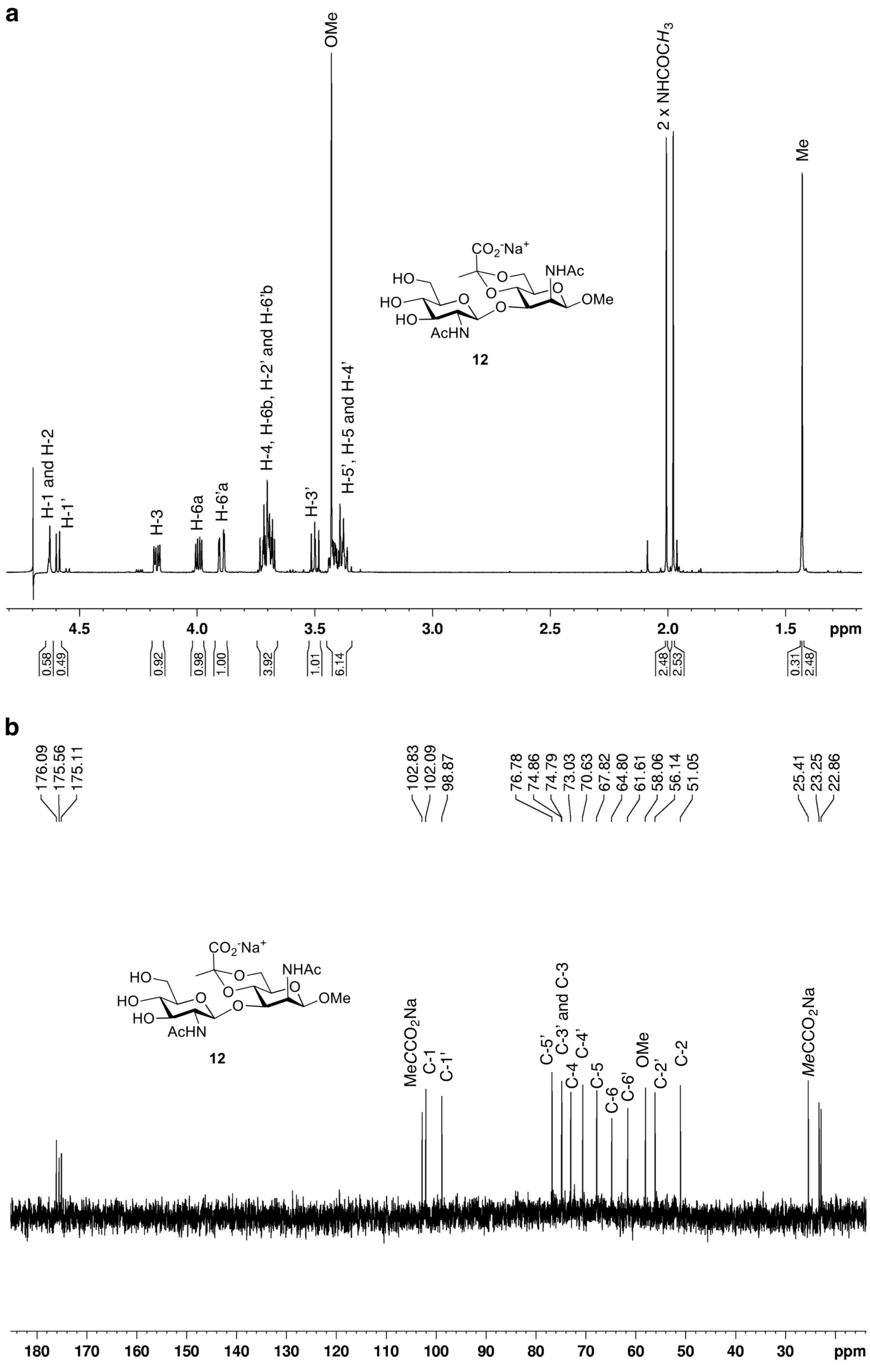

Supplementary Figure 4 | NMR spectra for compound 12. a, ^{1}H NMR spectrum (600 MHz) and **b,** ^{13}C NMR spectrum (600 MHz) of β-D-Glc*p*NAc-(1→3)-4,6-*O*-pyr-β-D-Man*p*NAcOMe (**12**) in D_2O.

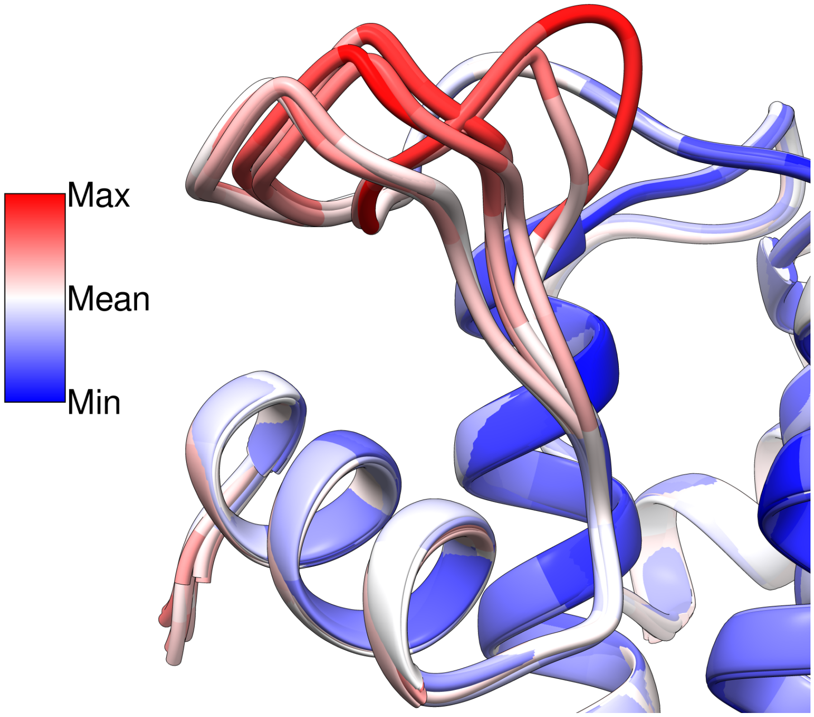

Supplementary Figure 5 | Multiple conformations of 44-55 loop. An overlay of ribbon diagrams of all crystallographically unique molecules of *wt* $SpaA_{SLH}$ is shown, focused on the connecting loop (residues 44-55) between the two helices of SLH1 beside G1. Ribbons are colored by average B-factor per residue, where white, red, and blue correspond to the mean, maximum, and minimum B-factor of each individual molecule.

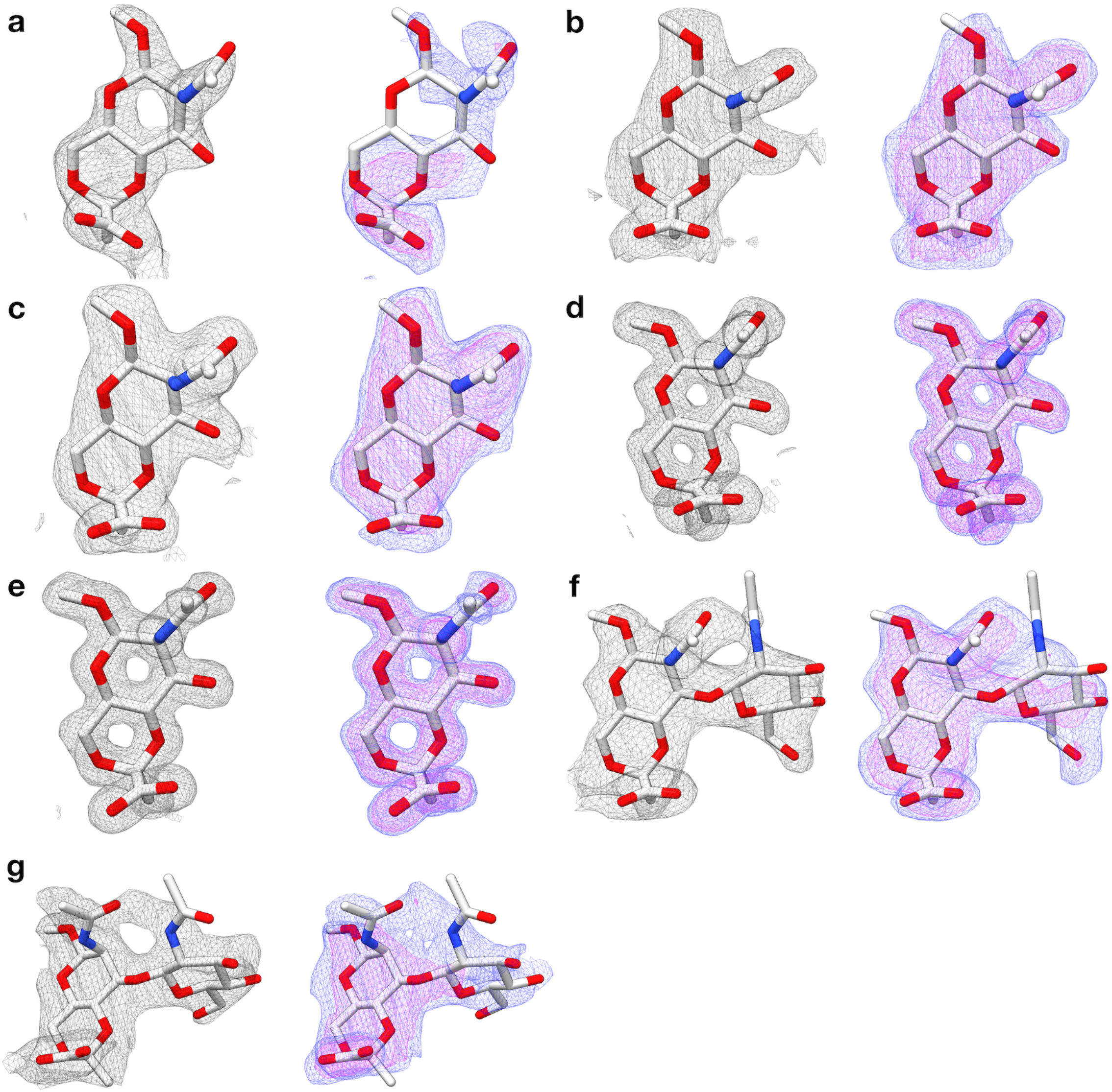

Supplementary Figure 6 | Electron density for bound ligands. 2Fo-Fc electron density maps contoured to 1σ (black) and omit Fo-Fc maps contoured to 2σ (blue) and 3σ (magenta) are shown for ligands bound in **a**, G1 of molecule A, **b**, G2 of molecule A, and **c**, G2 of molecule B of the structure of $SpaA_{SLH}$ in complex with monosaccharide (PDB 6CWI), **d**, G1 of the structure of $SpaA_{SLH}$/G109A in complex with monosaccharide (PDB 6CWN), **e**, G2 of the structure of $SpaA_{SLH}$/G46A/G109A in complex with monosaccharide (PDB 6CWR), and **f**, G2 of molecule A, and **g**, G2 of molecule B of the structure of $SpaA_{SLH}$ in complex with disaccharide (PDB 6CWL).

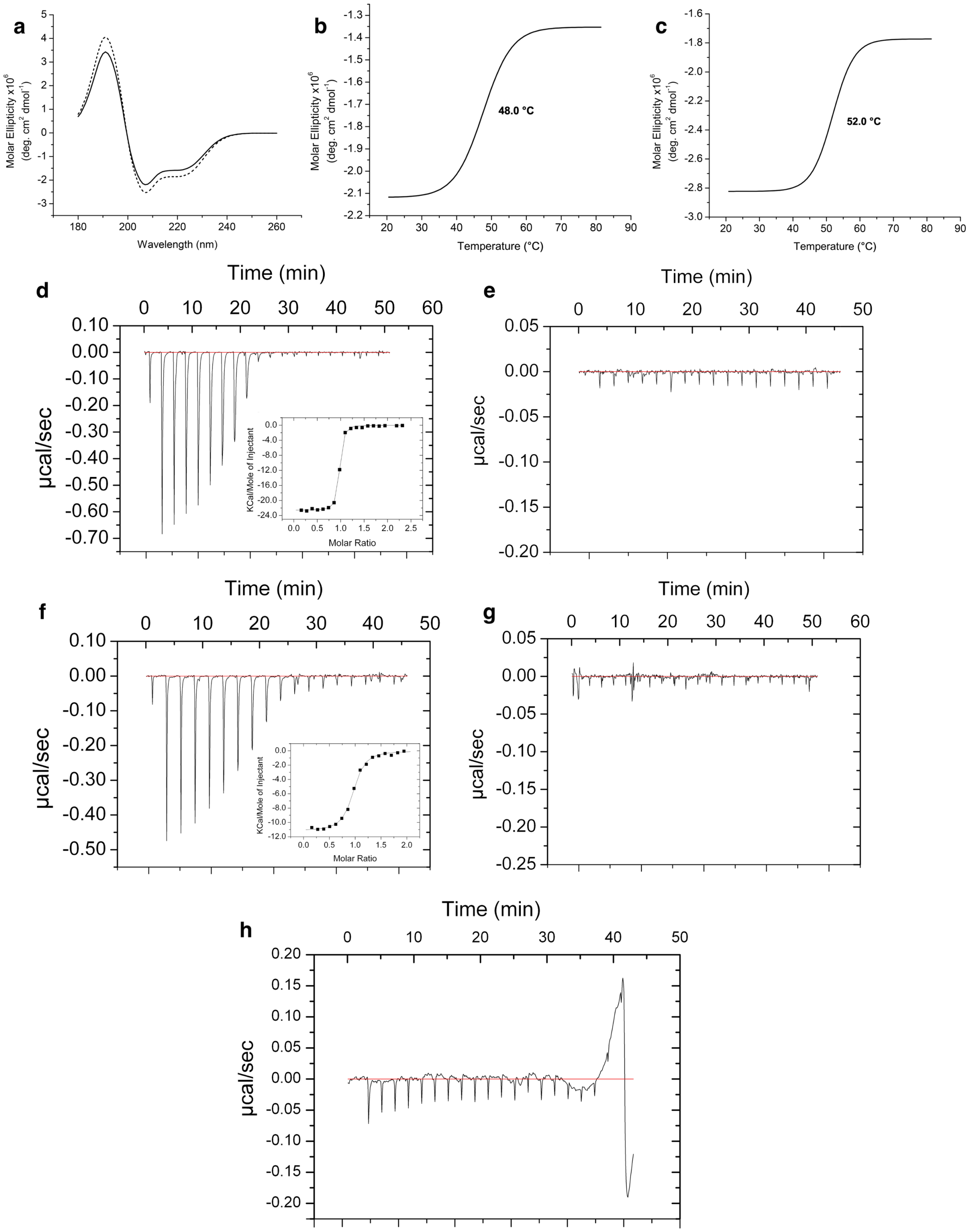

Supplementary Figure 7 | Biophysical analysis. a, Far-UV circular dichroism (CD) spectra are shown for $SpaA_{SLH}$ unliganded (continuous line) and complexed with monosaccharide (dashed line). Thermal denaturation spectra at 208 nm are shown for **b** unliganded $SpaA_{SLH}$ and **c** $SpaA_{SLH}$ complexed with monosaccharide. Binding isotherms from calorimetric titration are shown for $SpaA_{SLH}$ with synthetic **d** monosaccharide and **e** disaccharide ligands, of **f** $SpaA_{SLH}$/G109A and **g** $SpaA_{SLH}$/G46A/G109A with synthetic monosaccharide, and of **h** $SpaA_{SLH}$ with non-pyruvylated β-D-ManNAc as a control.

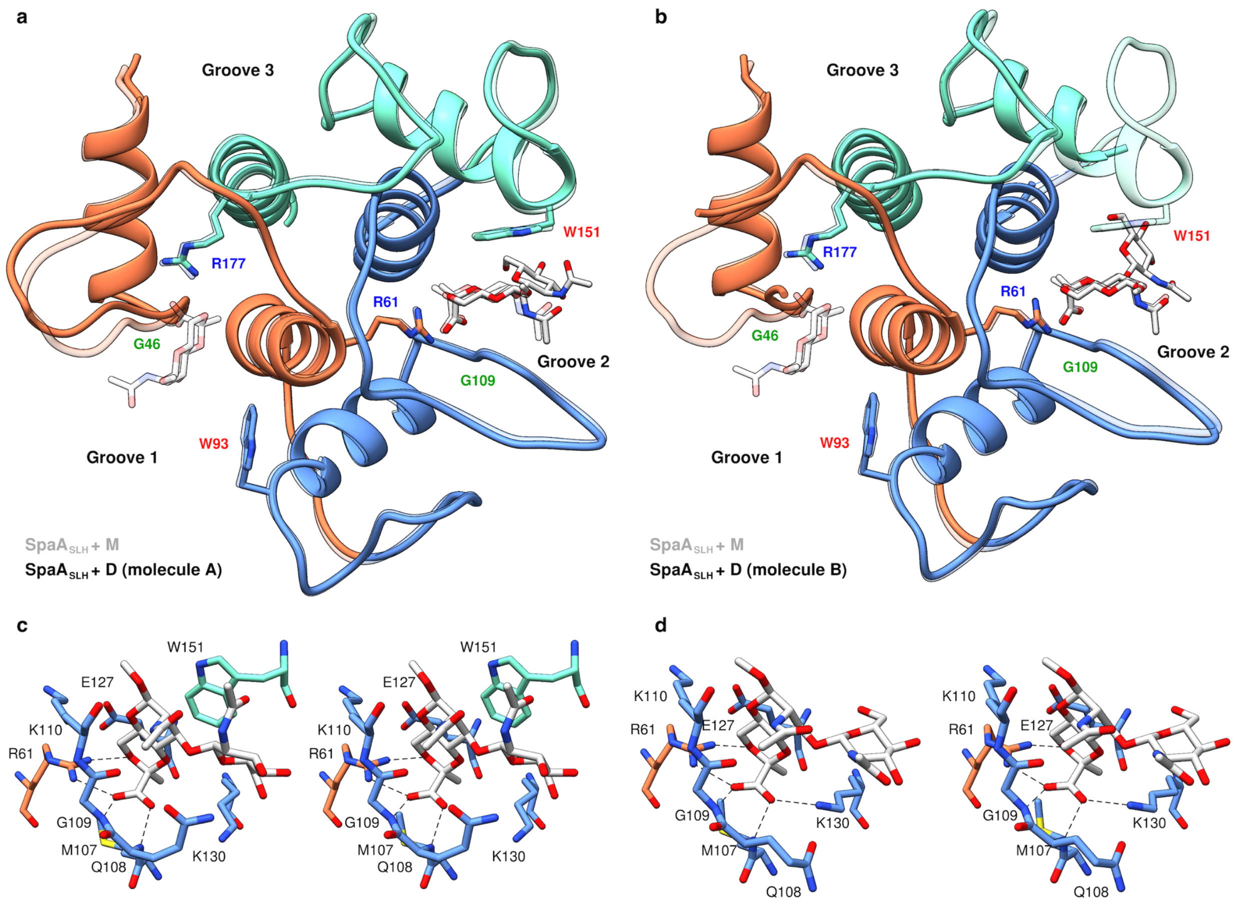

Supplementary Figure 8 | SpaA$_{SLH}$ binding to synthetic SCWP disaccharide β-D-GlcNAc-(1→3)-4,6-Pyr-β-D-ManNAcOMe. Ribbon diagrams of SpaA$_{SLH}$ with bound disaccharide (PDB 6CWL) are shown for molecules **a**, A and **b**, B of the structure, overlayed with the semi-transparent structure of SpaA$_{SLH}$ with bound monosaccharide (PDB 6CWI). Stereo views of disaccharide binding in G2 of molecules A and B are shown in **c** and **d**, respectively.

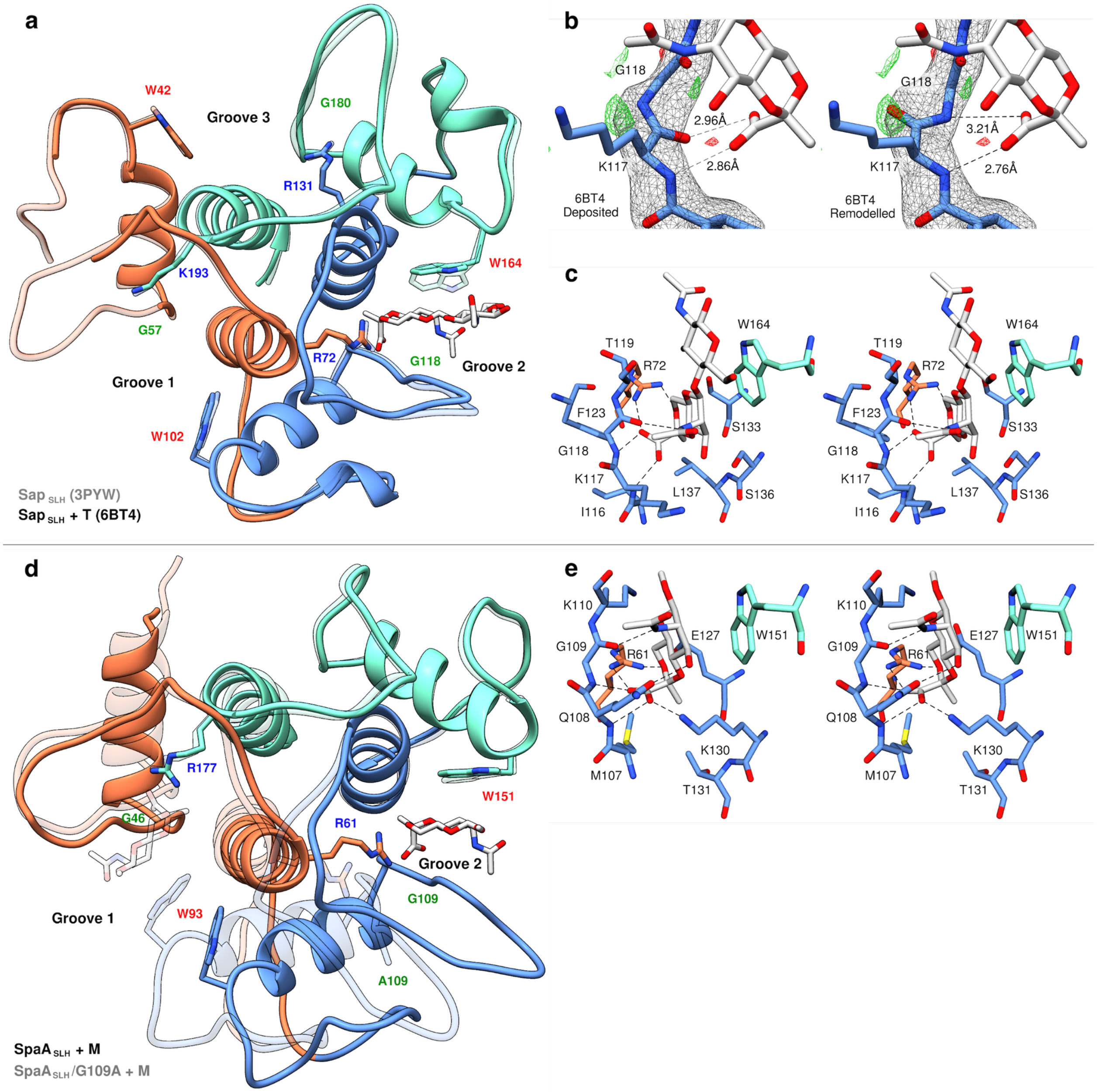

Supplementary Figure 9 | Comparison of *P. alvei* SpaA$_{SLH}$ and *B. anthracis* Sap$_{SLH}$. Ribbon diagrams of the unliganded and liganded crystal structures of Sap$_{SLH}$ are shown in **a**. The deposited electron density for liganded Sap$_{SLH}$ about Lys117 and G118 is shown in **b**, with the model as deposited, left panel, and remodelled with a flipped K117-G118 peptide bond to better fit the density, right panel (2Fo-Fc map contoured to 1σ, black; Fo-Fc map contoured to 3σ, green, and -3σ, red). A detailed stereo view of ligand bound in the remodelled G2 of Sap$_{SLH}$ is shown in **c**. For comparison, a ribbon diagram of SpaA$_{SLH}$ (PDB 6CWI) and SpaA$_{SLH}$/G109A (6CWN) with bound monosaccharides is shown in **d**, and a detailed stereo view of ligand bound in G2 of SpaA$_{SLH}$ is shown in **e**.

Supplementary Table 1A | Data collection and refinement statistics for wild-type $SpaA_{SLH}$

**Protein**	**$SpaA_{SLH}$**	**$SpaA_{SLH}$**	**$SpaA_{SLH}$**	**$SpaA_{SLH}$**	**$SpaA_{SLH}$**
**Ligand**	Unliganded	Monosaccharide[a]	Monosaccharide[a]	Monosaccharide[a]	Disaccharide[b]
**PDB code**	6CWC	6CWF	6CWH	6CWI	6CWL
**Data collection**					
Space group	$P3_221$	$P3_221$	P1	C2	$P3_221$
Resolution (Å)	40.0-1.90	40.0-2.25	40.0-2.00	40.0-2.15	40.0-2.15
	(1.93-190)[c]	(2.29-2.25)	(2.03-2.00)	(2.19-2.15)	(2.19-2.15)
Cell dimensions					
a (Å)	72.20	72.00	46.22	138.57	72.26
b (Å)	72.20	72.00	72.24	45.94	72.26
c (Å)	126.63	125.56	72.31	94.92	126.14
α (°)	90.00	90.00	86.73	90.00	90.00
β (°)	90.00	90.00	71.29	131.56	90.00
γ (°)	120.00	120.00	71.40	90.00	120.00
R_{sym}	0.072 (0.521)	0.047 (0.653)	0.044 (0.350)	0.046 (0.446)	0.073 (0.545)
R_{pim}	0.020 (0.135)	0.016 (0.222)	0.035 (0.273)	0.025 (0.265)	0.021 (0.152)
$CC_{1/2}$	(0.953)	(0.871)	(0.934)	(0.947)	(0.970)
I/σ(I)	33.3 (5.6)	44.8 (3.3)	19.9 (2.5)	27.1 (2.0)	34.3 (5.8)
Completeness (%)	99.6 (99.5)	100 (100)	95.0 (79.8)	97.1 (96.0)	99.9 (100)
Redundancy	14.7 (15.5)	9.4 (9.5)	2.4 (2.2)	4.3 (3.4)	13.2 (13.6)
Unique reflections	30690	18478	53737	23687	21339
**Refinement**					
Resolution	40.0-1.90	40.0-2.25	40.0-2.00	40.0-2.15	40.0-2.15
No. reflections	29054	17170	39929	17214	20168
R_{work} (%)	16.2	21.2	18.5	19.7	21.5
R_{free} (%)	21.4	25.4	23.5	23.7	25.5
No. atoms					
Protein	2544	2540	5096	2535	2393
Ligand	8 (Cl, SO_4)	42 (Ligand[a])	84 (Ligand[a])	73 (SO_4, Ligand[a])	70 (Ligand[b])
Water	212	90	350	126	83
B factors					
Protein	26.0	43.4	33.8	42.7	36.1
Ligand	46.1	42.6	24.7	42.5	53.0
Water	29.4	34.9	31.7	33.7	37.9
Ramachandran					
Favoured (%)	99	97	98	98	99
Allowed (%)	1	3	2	2	1
r.m.s. bonds (Å)	0.019	0.016	0.016	0.015	0.015
r.m.s. angles (°)	1.852	1.678	1.605	1.604	1.581

[a] Monosaccharide = 4,6-Pyr-β-D-ManNAcOMe;
[b] Disaccharide = β-D-GlcNAc-(1→3)-4,6-Pyr-β-D-ManNAcOMe.
[c] Values in parentheses represent the highest resolution shell.

Supplementary Table 1B | Data collection and refinement statistics for mutant SpaA$_{SLH}$

**Protein**	**SpaA$_{SLH}$/G109A**	**SpaA$_{SLH}$/G109A**	**SpaA$_{SLH}$/G46A/G109A**
**Ligand**	Unliganded	Monosaccharide[a]	Monosaccharide[a]
**PDB code**	6CWM	6CWN	6CWR
**Data collection**			
Space group	$P2_12_12_1$	C2	$P2_12_12_1$
Resolution (Å)	50.0-1.15 (1.17-1.15)[b]	50.0-1.53 (1.56-1.53)	30.0-1.24 (1.28-1.24)
Cell dimensions			
a (Å)	34.35	93.38	32.54
b (Å)	65.67	36.25	65.83
c (Å)	73.17	56.90	71.92
α (°)	90.00	90.00	90.00
β (°)	90.00	103.74	90.00
γ (°)	90.00	90.00	90.00
R_{sym}	0.043 (0.745)	0.044 (0.155)	0.040 (0.693)
R_{pim}	0.018 (0.303)	0.021 (0.097)	0.020 (0.473)
$CC_{1/2}$	(0.942)	(0.962)	(0.660)
I/σ(I)	38.4 (2.5)	28.6 (7.4)	27.2 (1.7)
Completeness (%)	96.4 (93.5)	98.6 (84.8)	99.2 (97.3)
Redundancy	7.3 (6.9)	4.3 (3.4)	4.1 (2.8)
Unique reflections	57497	27852	44339
**Refinement**			
Resolution	50.0-1.15	50.0-1.53	30.0-1.24
No. reflections	51418	26441	42050
R_{work} (%)	13.1	11.3	14.7
R_{free} (%)	15.9	15.1	17.9
No. atoms			
Protein	1337	1265	1284
Ligand	1 (Cl)	37 (MPD, Ligand[a])	21 (Ligand[a])
Water	185	176	166
B factors			
Protein	19.4	15.7	17.5
Ligand	26.4	33.0	17.7
Water	30.7	29.7	29.2
Ramachandran			
Favoured (%)	99	99	99
Allowed (%)	1	1	1
r.m.s. bonds (Å)	0.024	0.021	0.012
r.m.s. angles (°)	2.158	2.048	1.481

[a] Monosaccharide = 4,6-Pyr-β-D-ManNAcOMe
[b] Values in parentheses represent the highest resolution shell.

Supplementary Table 2 | SLH-28 and SLH-29 dihedral angles

Structure	SLH-28[a]		SLH-29[a]	
	phi (°)	psi (°)	phi (°)	psi (°)
Unliganded SpaA G1 (A)[b]	-123	160	-106	-150
Unliganded SpaA G1 (B)	-121	169	-54	163
Unliganded SpaA G2 (A)	-135	176	-70	164
Unliganded SpaA G2 (B)	-135	178	-72	163
Unliganded SpaA/G109A G1	-116	169	-62	160
Unliganded SpaA/G109A G2	-141	171	-55	149
Liganded SpaA G2 (A)[c]	-129	-19	86	-175
Liganded SpaA G2 (B)	-138	-5	89	-173
Liganded SpaA/G109A G1	-136	-8	62	-162

[a] SLH-28 corresponds to Ser45 in G1 and Gln108 in G2, and SLH-29 corresponds to Gly46 in G1 and Gly109 in G2.
[b] Letters in parentheses correspond to PDB chain ID.
[c] Dihedral angles are from the structure of $SpaA_{SLH}$ in complex with monosaccharide in space group C2 (PDB 6CWI).

Supplementary Table 3 | PCR amplification and DNA sequencing primers.

Primer	Sequence (5'→3') [a,b]	Comment
Fwd-SpaA$_{SLH}$-NdeI	ggctagCATATGGTAGCTTTCGGTGCTGACGCAGCAAAA ACAACTCAAGAGAAATTTGATGC	PCR amplification
Rev-SpaA$_{SLH}$-SacI	cgcgtcGAGCTCTTAGTGGTGGTGGTGGTGGTGAGAACC AGAACCTTTAGACATTTCGTCAGCTGCAAATGCTGC	PCR amplification
Fwd-mut-G109A(2.3)	GGCTTGATGCAA*GCC*AAAGATTTGACTAAG	Overlap extension PCR
Rev-mut-G109A(2.3)	CTTAGTCAAATCTTT*GGC*TTGCATCAAGCC	Overlap extension PCR
Fwd-mut-G46A(2.3)	GCTGGTGTATTCTCC*GCC*TATCCAGGAACTACT	Overlap extension PCR
Rev-mut-G46A(2.3)	AGTAGTTCCTGGATA*GGC*GGAGAATACACCAGC	Overlap extension PCR
Fwd-SlhA$_{SLH}$-NdeI	ggcgcgCATATGTACTCTTATCATGATGTGGTACAGCATC CT	PCR amplification
Rev-SlhA$_{SLH}$-XhoI	ggcgcgCTCGAGCATCTTAGGCAGTTTCTTCAAATCAATC AT	PCR amplification
Fwd-full-size SlhA double mutant/PvuII	TGAATCAGCTGTGGGACTTCCGTTA	PCR amplification
Rev-full-size SlhA double mutant/KpnI	agagtcGGTACCTTACATCTTAGGCAGTTTCTT	PCR amplification
Fwd-mut-G1224A(2.3)	GAAGGTATCGTCCGT*GCC*GTATCGCCGAATGAATTTGG C	Overlap extension PCR
Rev-mut-G1224A(2.3)	GCCAAATTCATTCGGCGATAC*GGC*ACGGACGATACCT TC	Overlap extension PCR
Fwd-mut-G1293A(3.2)	GAAGAAAGGCTTTATTTCG*GCG*GTTCCAATCGATCCTT CT	Overlap extension PCR
Rev-mut-G1293A(3.2)	AGAAGGATCGATTGGAAC*CGC*CGAAATAAAGCCTTTC TTC	Overlap extension PCR

[a] Restriction sites are underlined. Lower case letters indicate artificially introduced bases to improve restriction enzyme cutting.

[b] Mutating codons are underlined and shown in italic.

Supplementary References

1. Murphy, P. V., O'Brien, J. L., Gorey-Feret, L. J. & Smith, A. B. Synthesis of novel HIV-1 protease inhibitors based on carbohydrate scaffolds. *Tetrahedron* **59,** 2259–2271 (2003).
2. Ellervik, U. & Magnusson, G. Glycosylation with N-Troc-protected glycosyl donors. *Carbohydr. Res.* **280,** 251–260 (1996).
3. Schüle, G. & Ziegler, T. Efficient convergent block synthesis of a pyruvated tetrasaccharide 5-aminopentyl glycoside related to *Streptococcus pneumoniae* type 27. *Tetrahedron* **52,** 2925–2936 (1996).
4. Jansson, P.-E., Lindberg, J. & Widmalm, G. Syntheses and NMR studies of pyruvic acid 4, 6-acetals of some methyl hexopyranosides. *Acta Chem Scand* **47,** 711–715 (1993).
5. Augé, J., David, S., Guibe, L. & Jugie, G. A nuclear-quadrupole resonance study of halogen substituted methyl pyranosides-dependence of the p-orbital population of a halogen at position 2 upon the anomeric configuration and a possible correlation with chemical reactivity. *Noveau J. Chim* **4,** 481–486 (1980).
6. Kern, J. *et al.* Structure of surface layer homology (SLH) domains from *Bacillus anthracis* surface array protein. *J. Biol. Chem.* **286,** 26042–26049 (2011).
7. Sychantha, D. *et al.* Molecular Basis for the Attachment of S-Layer Proteins to the Cell Wall of *Bacillus anthracis*. *Biochemistry* (2018). doi:10.1021/acs.biochem.8b00060
8. Rünzler, D., Huber, C., Moll, D., Köhler, G. & Sára, M. Biophysical characterization of the entire bacterial surface layer protein SbsB and its two distinct functional domains. *J. Biol. Chem.* **279,** 5207–5215 (2004).
9. Pavkov, T. *et al.* The structure and binding behavior of the bacterial cell surface layer protein SbsC. *Structure* **16,** 1226–1237 (2008).
10. Janesch, B., Messner, P. & Schäffer, C. Are the surface layer homology domains essential for cell surface display and glycosylation of the S-layer protein from Paenibacillus alvei CCM 2051^{T}? *J. Bacteriol.* **195,** 565–75 (2013).
11. May, A., Pusztahelyi, T., Hoffmann, N., Fischer, R.-J. & Bahl, H. Mutagenesis of conserved charged amino acids in SLH domains of *Thermoanaerobacterium thermosulfurigenes* EM1 affects attachment to cell wall sacculi. *Arch. Microbiol.* **185,** 263–269 (2006).
12. Zarschler, K. *et al.* Protein tyrosine *O*-glycosylation - a rather unexplored prokaryotic glycosylation system. *Glycobiology* **20,** 787–98 (2010).
